# Integrin α5β1, as a Receptor of Fibronectin, Binds the FbaA Protein of Group A *Streptococcus* To Initiate Autophagy during Infection

**DOI:** 10.1128/mBio.00771-20

**Published:** 2020-07-16

**Authors:** Jiachao Wang, Meiqi Meng, Miao Li, Xiaofei Guan, Jianguo Liu, Xue Gao, Qingqing Sun, Jinquan Li, Cuiqing Ma, Lin Wei

**Affiliations:** a Department of Immunology, Key Laboratory of Immune Mechanism and Intervention on Serious Disease in Hebei Province, Hebei Medical University, Shijiazhuang, China; b Departments of Internal Medicine & Molecular Microbiology and Immunology, Saint Louis University School of Medicine, St. Louis, Missouri, USA; c State Key Laboratory of Agricultural Microbiology, College of Food Science and Technology, Huazhong Agricultural University, Wuhan, Hubei, China; College of Veterinary Medicine, Cornell University

**Keywords:** autophagy, group A *Streptococcus*, FbaA protein, integrin α5β1, fibronectin

## Abstract

Group A *Streptococcus* (GAS), one of the most common extracellular pathogens, has been reported to invade epithelial and endothelial cells. Our results reveal that M1 GAS strain SF370 can be effectively eliminated by respiratory epithelial cells. Emerging evidence indicates that autophagy is an important strategy for nonphagocytes to eliminate intracellular bacteria. Upon pathogen recognition, cell surface receptors can directly trigger autophagy, which is a critical step in controlling infection. However, the mechanisms of how cells sense invading bacteria and use this information specifically to trigger autophagy remain unclear. In this study, we stimulated cells and infected mice with M and FbaA mutants of M1 GAS strain SF370 or with purified M and FbaA proteins (two critical surface structural proteins of GAS), and found that only FbaA protein was involved in autophagy induction. Furthermore, the FbaA protein induced autophagy independent of common pattern recognition receptors (such as Toll-like receptors); rather, it relies on binding to integrin α5β1 expressed on the cell surface, which is mediated by extracellular matrix protein fibronectin (Fn). The FbaA-Fn-integrin α5β1 complex activates Beclin-1 through the mTOR-ULK1–Beclin-1 pathway, which enables the Beclin-1/Vps34 complex to recruit Rab7 and, ultimately, to promote the formation of autophagosomes. By knocking down integrin α5β1, Fn, Atg5, Beclin-1, and ULK1 in Hep2 cells and deleting Atg5 or integrin α5β1 in mice, we reveal a novel role for integrin α5β1 in inducing autophagy. Our study demonstrates that integrin α5β1, through interacting with pathogen components, initiates effective host innate immunity against invading intracellular pathogens.

## Abstract (precis)

Autophagy is generally considered a strategy used by the innate immune system to eliminate invasive pathogens through capturing and transferring them to lysosomes. Currently, researchers pay more attention to how virulence factors secreted by GAS regulate the autophagic process. Here, we provide the first evidence that the structural protein FbaA of M1 GAS strain SF370 is a potent inducer of autophagy in epithelial cells. Furthermore, we demonstrate that integrin α5β1 in epithelial cells *in vitro* and *in vivo* acts as a receptor to initiate the signaling for inducing autophagy by binding to FbaA of M1 GAS strain SF370 via Fn. Our study reveals the underlying mechanisms by which pathogens induce Fn-integrin α5β1 to trigger autophagy in a conserved pattern in epithelial cells.

## INTRODUCTION

Group A *Streptococcus* (GAS; Streptococcus pyogenes) is one of the most common and diverse human pathogens and symbiotic organisms (1). GAS can cause a wide range of mild to severe infections in almost every organ in people of all ages, especially in the elderly and children. Each year, at least 517,000 people worldwide die from complications of severe GAS infection, including streptococcal toxic shock syndrome, life-threatening bacteremia, and necrotizing fasciitis (2). Therefore, understanding the pathogenesis and exploring new treatment strategies for GAS-associated diseases are critical to reduce the burden. Traditionally, GAS is treated as an extracellular bacterium. However, increasing evidence has shown that GAS can invade epithelial and endothelial cells through GAS structural proteins (such as the M protein, FbaA protein, etc.) (3, 4) or secreted proteins (such as streptolysin O, streptococcal pyrogen exotoxin B, etc.) to escape the immune defense mechanism of the host (5).

Autophagy is an evolutionarily conserved and lysosome-dependent protein degradation pathway in almost all types of cells. Canonical autophagy is characterized by the dynamic formation of a bilayer membrane structure called the autophagosome (6). The main function of basal autophagy is to degrade long-lived cellular proteins and damaged organelles for recycling (7). Autophagy can also selectively target unwanted materials within cells, including invading microbes. Autophagy that targets bacteria is called xenophagy (8). A growing amount of evidence suggests that xenophagy is an important innate immune protective mechanism in cells (9).

The cytoplasm is the battlefield for survival between host and pathogen, and the life-and-death fight is initiated by host-pathogen interactions, such as autophagy induction. The M1T1^5448^ GAS serotype replicates efficiently in the cytoplasm of infected cells by degrading autophagy adaptor proteins P62, NBR1, and NDP52 through secreted protein SpeB (10). However, it has been reported that some GAS strains, such as M6, M49, and M89, are effectively internalized into epithelial cells, where they are targeted for autophagy and then cleared (11, 12). After GAS invades cells, streptococcal hemolysin O (SLO) contributes to the escape of GAS from endosomes and its entry into the cytoplasm. At least one-half of cytoplasmic GAS bacteria colocalize with ubiquitin and P62, suggesting that cytoplasmic GAS are targeted for autophagy (13). Gal-8 (galectin-8) is highly expressed in epithelial cells, binds to the surface of GAS or GAS-containing ruptured phagosomes, and recruits Parkin (an E3 ligase), which is subsequently involved in polyubiquitination of GAS or GAS-containing phagosomes (Lys63-linked) (14). Accordingly, polyubiquitination leads to the recruitment of the autophagy adaptor proteins NDP52, P62, and NDR1 and then triggers LC3 recruitment, which promotes autophagosome formation. GAS enters the cytoplasm to form LC3II-positive GAS-rich autophagosome-like vacuoles (GcAV), not conventional autophagosomes (15), and multiple Rab GTPases were involved in the formation of GcAVs, including Rab1 (16), Rab9A, Rab23 (17), and Rab30 (18). In phagocytic cells, NO and ROS induce autophagy by promoting cGMP nitration to form 8-nitro-cGMP, which accelerates the autophagic clearance of cytoplasmic GAS that has been modified by the Lys63-linked ubiquitin chain (19). However, these studies focused on the process of intracellular GAS-induced autophagy. Whether extracellular GAS is a direct initiator of autophagy has not been reported. We found that heat-inactivated M1 GAS strain SF370 can also induce autophagy, suggesting a role for bacterial structural proteins in autophagy induction. Thus, we constructed surface protein-defective strains of M1 GAS strain SF370 and found that FbaA was a strong autophagy inducer. It has been reported that bacteria can initiate autophagy through Toll-like receptors (TLRs) (20). However, our experiments demonstrated that FbaA was able to induce autophagy in cells without TLR2 and TLR4. FbaA is a novel Fn binding protein that can bind to Fn, which then binds to integrin α5β1 on the surface of host cells during pathogen invasion (21). Whether the GAS-cell interaction can trigger autophagy is unclear. As an extracellular matrix (ECM) component, Fn is secreted by a series of host cells and can bind to bacterial FnBP and to its receptors, integrin molecules on the host cell surface, such as integrin α5β1. Thus, Fn acts as a bridge that links pathogens to the surface of the host cells during invasion. Here, we show that the molecular mechanism of autophagy induction involves the interaction between FbaA, Fn, and integrin α5β1 on the host cell surface. This study reveals the first direct visualization of integrin α5β1, as a receptor commonly expressed on nonphagocytes, during autophagy initiation.

## RESULTS

### Autophagy contributes to M1 GAS strain SF370 elimination from invaded cells.

**FIG 1:**
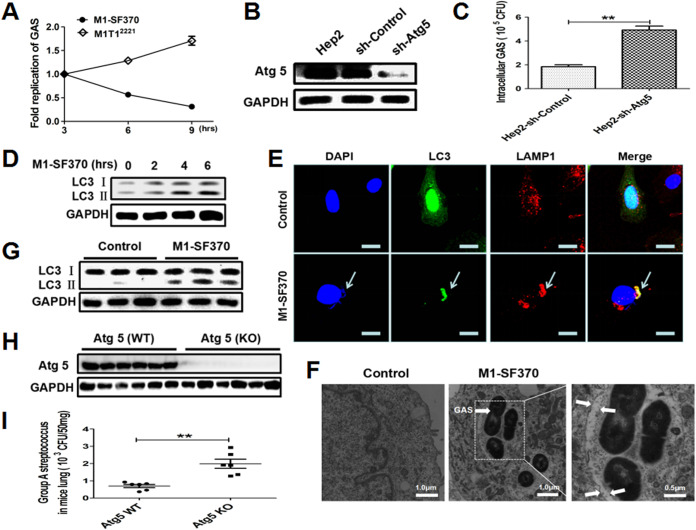
M1 GAS strain SF370 induces autophagy *in vivo* and *in vitro*. (A) Hep-2 cells were infected with M1 GAS strain SF370 or M1TI^2221^ GAS (MOI, 100:1). Shown are the numbers of CFU of viable bacteria in Hep2 cells at different time points. (B) The silencing efficiency of Atg5 shRNA (sh-Atg5) was validated by Western blotting with anti-Atg5 antibody. GAPDH served as a control. (C) The replication ability of the M1 GAS strain SF370 invaders in sh-Atg5-Hep2 cells and sh-control Hep2 cells was determined 6 h following infection. (D) LC3II expression was analyzed by Western blotting of proteins from M1 GAS strain SF370-infected Hep2 cells. (E) After Hep2 cells were transfected with the pBABE-puro-EGFP-LC3 plasmid for 24 h and then infected with M1 GAS strain SF370, the host cell and M1 GAS strain SF370 DNA were stained with DAPI, and the presence of fluorescent EGFP-LC3 puncta and LAMP1 was determined by confocal laser scanning microscopy. Scale bar, 25 μm. White arrows refer to M1 GAS strain SF370. (F) Presence of autophagosomes in M1 GAS strain SF370-infected Hep2 cells was observed by a transmission electron microscope. White arrows refer to double-membraned autophagosomes. (G) Results from the Western blot analysis of LC3II expression in the lungs of mice i.n. infected with M1 GAS strain SF370 bacteria (3 × 10^8^ CFU) or PBS as a control. (H and I) Atg5 (KO) in the mice was confirmed by Western blotting. (H) Cells were infected i.n. with M1 GAS strain SF370 bacteria (3 × 10^8^ CFU). (I) After 24 h, the mouse lungs were harvested to detect the number of viable GAS bacteria by CFU count. Data are presented as the means ± SD from three independent experiments *in vitro* with at least 6 mice per group. *, *P *< 0.05; **, *P* < 0.01.

Hep2 cells were used to evaluate the fate of the M1 GAS strain SF370 or M1TI^2221^ GAS infection in epithelial cells. After infection, the number of viable M1 GAS strain SF370 CFU inside the cells gradually decreased (Fig. 1A), while host cell viability was not changed (data not shown). In contrast, M1TI^2221^ GAS can not only survive but also replicate within epithelial cells. These results suggest that not all GAS strains can evade immune defenses and replicate effectively in epithelial cells. Therefore, how epithelial cells clear the invading M1 GAS strain SF370 is unclear. Recently, autophagy was recognized as an important component of the innate immune system, contributing significantly to cellular homeostasis. To decide whether autophagy is involved in the clearance of M1 GAS strain SF370 in Hep2 cells, we constructed an autophagy-defective cell line by knocking down Atg5 (a key protein for autophagy) (22) (Fig. 1B) and found that the number of viable intracellular bacteria in Atg5-deficient cells was much higher than that in autophagy-intact cells after M1 GAS strain SF370 infection (Fig. 1C). These data suggest a role for autophagy in the clearance of M1 GAS strain SF370 in Hep2 cells. Indeed, the level of LC3II was increased significantly 4 h after M1 GAS infection (Fig. 1D). To determine whether the M1 GAS strain SF370 pathogens were cleared by autolysosomes, we used confocal microscopy to determine their colocalization with LC3 and LAMP1 (a lysosomal membrane marker) in M1 GAS strain SF370-infected cells. The confocal microscopy evidence indicated that M1 GAS strain SF370 infection changed the distribution of enhanced green fluorescent protein (EGFP)-LC3 from being diffuse to aggregating as puncta, and M1 GAS strain SF370, LC3, and LAMP1 were colocalized (Fig. 1E). Subsequently, a typical bilayer vacuole containing M1 GAS strain SF370 was observed in the cytoplasm of M1 GAS strain SF370-infected Hep2 cells by transmission electron microscopy (TEM) (Fig. 1F), indicating the formation of autophagosomes. In the mouse model, the results were consistent with those of human cells, in that the expression of LC3II was increased in the homogenized M1 GAS strain SF370-infected lungs compared with its expression in the mice treated with the phosphate-buffered saline (PBS) control (Fig. 1G). To confirm the function of autophagy in the clearance of M1 GAS strain SF370 in the lung, we infected Atg5-deficient (knockout [KO]) mice with M1 GAS strain SF370 (Fig. 1H), and the results showed that the number of viable bacteria in the lungs of Atg5-KO mice was much higher than it was in the lungs of wild-type (WT) mice (Fig. 1I). Taken together, these results suggest that autophagy mediates the clearance of intracellular M1 GAS strain SF370 in epithelial cells.

### M1 GAS strain SF370 surface protein FbaA mediates autophagy induction.

**FIG 2:**
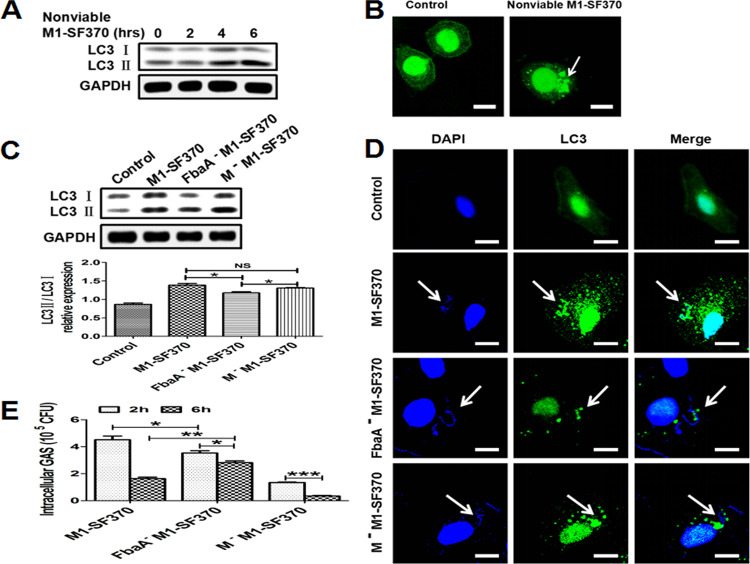
Evaluation of autophagy induction by M1 GAS strain SF370 proteins. (A and B) Hep2 cells were infected with nonviable M1 GAS strain SF370 for the indicated times, and LC3 expression was assessed by Western blotting (A) or confocal laser scanning microscopy (B). Scale bar, 25 μm. (C to E) Hep2 cells were infected with WT M1 GAS strain SF370, FbaA^−^M1 GAS strain SF370, or M^−^M1 GAS strain SF370. (C) LC3II expression was analyzed by Western blotting. (D) The colocalization of fluorescent EGFP-LC3 puncta and M1 GAS strain SF370 (DAPI staining) was observed by confocal laser scanning microscopy. Scale bar, 25 μm. (E) Numbers of CFU of WT M1 GAS strain SF370, FbaA^−^M1 GAS strain SF370, and M^−^M1 GAS strain SF370 within Hep2 cells were counted after infection for the indicated times. *, *P *< 0.05; **, *P* < 0.01; ***, *P* < 0.001.

**FIG 3:**
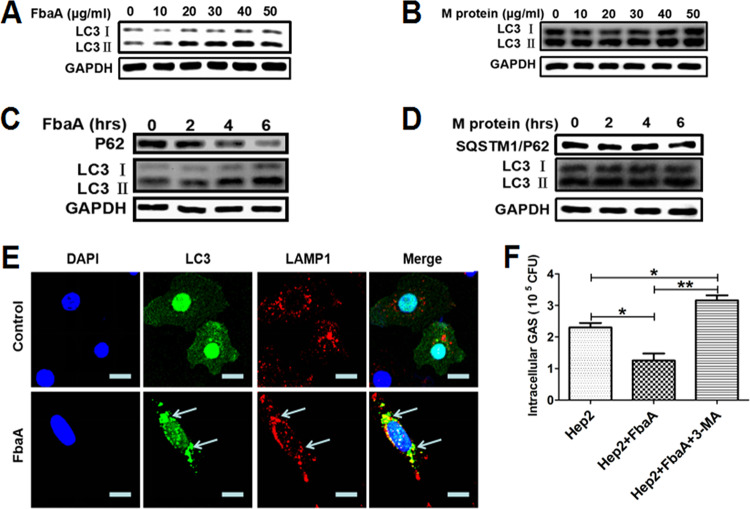
FbaA stimulation alone induces complete autophagic flux, which facilitates the elimination of intracellular M1 GAS strain SF370. (A and B) Hep2 cells were stimulated with the M or FbaA protein. LC3 expression in the Hep2 cells stimulated with different concentrations of FbaA (A) or M protein (B) for 6 h and analyzed by Western blotting. (C and D) Expression of LC3II and P62 in Hep2 cells stimulated with 30 μg/ml FbaA (C) or M protein (D) for the indicated times, as assessed by Western blotting. (E) A confocal laser scanning microscope was used to observe fluorescent EGFP-LC3 puncta and LAMP1 in Hep2 cells following FbaA stimulation for 6 h. Scale bar, 25 μm. (F) Hep2 cells were pretreated with purified FbaA protein (30 μg/ml) or FbaA combined with 3-MA (10 mM) for 0.5 h and then infected with M1 GAS strain SF370 for 6 h. Intracellular M1 GAS strain SF370 CFU were detected in cells. *, *P *< 0.05; **, *P* < 0.01.

The SpeB protein produced by M1T1 GAS has secretory and enzymatic activity and plays a key role in regulating autophagy. However, whether M1 GAS strain SF370-induced autophagy is associated with secretory enzyme proteins is relatively unknown. We measured the expression of autophagy-related protein LC3 in Hep2 cells stimulated with heat-inactivated M1 GAS strain SF370 and found that LC3II was highly expressed at 4 h after stimulation (Fig. 2A). Confocal microscopy evidence also showed an increase in EGFP-LC3 puncta in the cytoplasm (Fig. 2B), indicating autophagy was induced by inactivated M1 GAS strain SF370. These results suggest that the protein structure of the M1 GAS strain SF370 is the key to inducing autophagy. The M and FbaA proteins are known to be the main bacterial structural proteins of M1 GAS strain SF370. Therefore, we infected Hep2 cells with strains of M1 GAS strain SF370 deficient in these proteins (FbaA^−^M1 GAS strain SF370 and M^−^M1 GAS strain SF370) and with WT M1 GAS strain SF370 and found that WT M1 GAS strain SF370- and M^−^M1 GAS strain SF370-infected cells induced higher levels of the LC3II protein than the FbaA^−^M1 GAS strain SF370-infected cells (Fig. 2C). A similar result was shown by confocal microscopy (Fig. 2D). Next, we determined survival of the three strains in Hep2 cells after infection. At 2 h after infection, we found that the intracellular survival rate of FbaA^−^M1 GAS strain SF370 and M^−^M1 GAS strain SF370 was lower than that of WT M1 GAS strain SF370, indicating that the FbaA protein and M protein were involved in the invasion of M1 GAS strain SF370, mainly the M protein (Fig. 2E). Six hours after infection, the results showed that M^−^M1 GAS strain SF370 had the lowest intracellular viability of these three strains, while FbaA^−^M1 GAS strain SF370 had the highest intracellular viability (Fig. 2E). These results indicate that the FbaA protein but not the M protein is associated with M1 GAS strain SF370-induced autophagy. Our previous studies also showed that the M and FbaA proteins facilitated M1 GAS strain SF370 invasion of host cells (23). In line with these results, a lack of M protein (M^−^M1 GAS strain SF370) led to reduced levels of bacterial invasion. This low invasion rate may be the reason that more intracellular WT M1 GAS strain SF370 than M^−^M1 GAS strain SF370 was detected in cells at 6 h after infection (Fig. 2E), despite the fact that they all induced high levels of LC3II expression. To confirm that M1 GAS strain SF370-induced autophagy is associated with the FbaA protein but not the M protein, Hep2 cells were stimulated separately with different concentrations of FbaA and M proteins and then LC3 expression was measured. The results showed that the expression of LC3II was increased in a dose-dependent manner by the FbaA protein (Fig. 3A) but not by the M protein (Fig. 3B). In subsequent experiments, we chose a concentration of 30 μg/ml to clearly demonstrate FbaA-induced autophagy *in vitro*. FbaA-induced autophagy peaked at approximately 6 h (Fig. 3C), while the M protein again had no effect on LC3 expression (Fig. 3D). In addition, confocal microscopy also observed an increase in EGFP-LC3 protein puncta aggregation after FbaA stimulation and EGFP-LC3 colocalized with LAMP1 (Fig. 3E). The evaluation of complete autophagy generally includes two aspects: first, the formation of autophagosomes, and second, autophagosome and lysosome barrier-free fusion and degradation of their contents. Currently, SQSTM1/P62 (a ubiquitin-binding protein) is a protein marker that responds to autophagic flux. During autophagy formation, P62 binds to the ubiquitinated protein and forms a complex with the LC3II protein localized on the inner membrane of an autophagosome and is then degraded in the autophagolysosome (24). P62 levels were reduced after Hep2 cells were treated with FbaA protein (Fig. 3C), suggesting that the FbaA protein induces complete autophagic flux. To further confirm the role of FbaA-induced autophagy in the clearance of M1 GAS strain SF370, the Hep2 cells were pretreated with purified FbaA protein and/or 3-MA (3-methyladenosine; an autophagy inhibitor) for 0.5 h and then infected with M1 GAS strain SF370 for 6 h, followed by counting the number of viable bacteria in the cells. The results showed that FbaA-induced autophagy significantly increased the clearance of M1 GAS strain SF370, and this increased clearance was blocked by 3-MA (Fig. 3F). Taken together, our results demonstrate that M1 GAS strain SF370 induces autophagy through the bacterial structure protein FbaA but not the M protein.

### The domain from amino acids 68 to 161 in the FbaA protein mediates autophagy induction.

**FIG 4:**
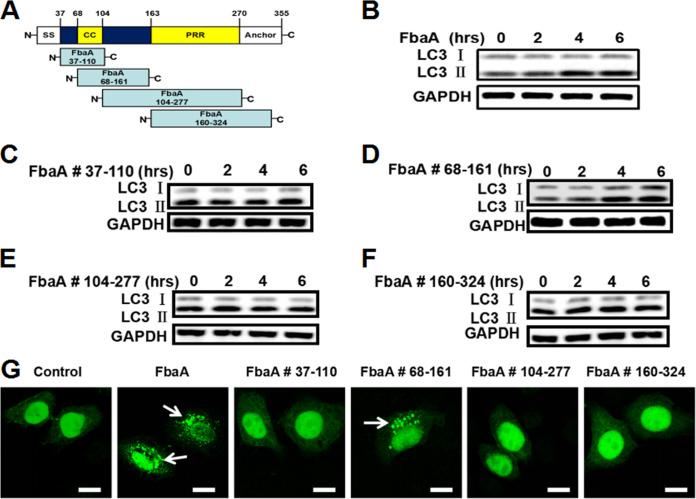
Amino acids 68 to 161 of the FbaA protein induce autophagy. (A) Schematic representation of FbaA and FbaA derivative constructs. The structural and functional domains of FbaA are the following: signal sequence (SS); coiled-coil region (CC); proline-rich repeats (PRR); and anchor, cell wall, and membrane-anchoring region. (B to G) Hep2 cells were stimulated with FbaA, FbaA #37-110, FbaA #68-161, FbaA #104-277, or FbaA #160-324 for the indicated times. (B to F) LC3II expression as determined by Western blotting. (G) Fluorescent EGFP-LC3 puncta, as determined by confocal laser scanning microscopy. Scale bar, 25 μm.

To further clarify the domain in FbaA protein critical for autophagy induction, we constructed four functional FbaA peptides using Escherichia coli BL-21 according to the structure of FbaA (25). The four peptides were FbaA #37-110, FbaA #68-161, FbaA #104-277, and FbaA #160-324 (Fig. 4A). The Western blot analysis results showed that only FbaA #68-161 induced high levels of LC3II expression (Fig. 4D), similar to FbaA protein stimulation (Fig. 4B), and neither FbaA #37-110, FbaA #104-277, nor FbaA #160-324 had an effect on autophagy induction (Fig. 4C, E, and F). The confocal microscopy also showed the enhanced aggregation of EGFP-LC3 puncta induced by the FbaA and FbaA #68-161 treatments (Fig. 4G). It is worth noting that FbaA #68-161 contains the coiled-coil region, and the primary function of the region is to mediate protein interactions. FbaA #37-110 also contains the coiled-coil region but fails to induce autophagy, suggesting that the coiled-coil region alone is not sufficient to induce autophagy. It has been reported that multiple, variable N termini of FbaA function to bind repeat domains, and the C terminus is the conserved structure (26). The amino acid sequence of FbaA #68-161 contains a coiled-coil region and a partial fragment of the N terminus, which may be required for inducing autophagy. The specific structural features of the amino acid sequence involved in inducing autophagy have yet to be studied.

### Reconstitution of FbaA in FbaA^−^ M1 GAS strain SF370.

**FIG 5:**
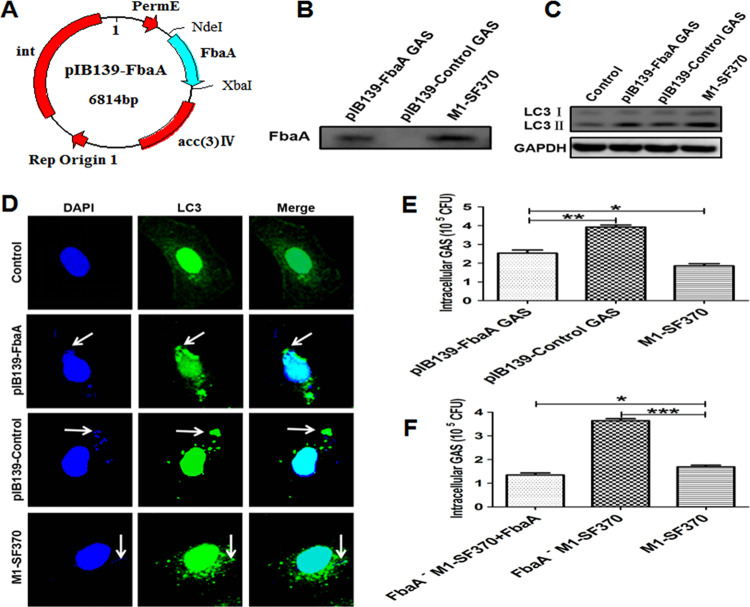
Reconstitution of FbaA in FbaA^−^M1 GAS strain SF370 and the evaluation of autophagy induction. (A) Map of the constructed pIB139-FbaA plasmid. (B) Membrane proteins from the WT M1 GAS strain SF370, pIB139-Control GAS, and pIB139-FbaA GAS were extracted. The expression of FbaA was detected by Western blotting. (C) Hep2 cells were infected with WT M1 GAS strain SF370, pIB139-Control GAS, or pIB139-FbaA GAS, and the expression of LC3II was analyzed by Western blotting. (D) Colocalization of fluorescent EGFP-LC3 puncta with WT M1 GAS strain SF370, pIB139-Control GAS, and pIB139-FbaA GAS (DAPI staining) was observed by confocal laser scanning microscopy. Scale bar, 25 μm. (E) Numbers of CFU of WT M1 GAS strain SF370, pIB139-Control GAS, and pIB139-FbaA GAS bacteria within Hep2 cells were counted at 6 h after infection. (F) Hep2 cells were pretreated with purified FbaA protein (30 μg/ml) for 0.5 h and then infected with WT M1 GAS strain SF370 or FbaA^−^M1 GAS strain SF370 for 6 h. CFU of intracellular GAS in the cells were detected. *, *P *< 0.05; **, *P* < 0.01; ***, *P* < 0.001.

The above-described experiments indicate that the structural protein FbaA in M1 GAS strain SF370 is the main component for autophagy induction. To determine whether autophagy was indeed triggered by FbaA, FbaA^−^M1 GAS strain SF370 needed to be complemented and the phenotype restored to rule out any possibility of secondary mutation. We inserted the FbaA gene into the pIB139 shuttle integrative vector and then reconstituted FbaA into the FbaA^−^M1 GAS strain SF370 by electroporation to obtain the gene recovery strain, named pIB139-FbaA GAS, and pIB139-Control GAS (Fig. 5A). The FbaA membrane proteins in WT M1 GAS strain SF370, pIB139-Control GAS, and pIB139-FbaA GAS were extracted, and the Western blotting results suggested that FbaA was reexpressed on the surface of FbaA- M1 GAS strain SF370 (Fig. 5B). To determine whether pIB139-FbaA GAS could successfully induce autophagy, Hep2 cells were infected with WT M1 GAS strain SF370, pIB139-Control GAS, or pIB139-FbaA GAS for 6 h. The results showed that pIB139-FbaA GAS induced higher levels of LC3II protein expression than pIB139-Control GAS-infected cells (Fig. 5C). Confocal microscopy also showed an increase in EGFP-LC3 puncta in the cytoplasm of the pIB139-FbaA GAS-infected cells (Fig. 5D). In addition, the number of intracellular viable pIB139-FbaA GAS bacteria was much lower than it was in the pIB139-Control GAS-infected cells (Fig. 5E). Similarly, the addition of exogenous FbaA protein restored FbaA^−^M1 GAS strain SF370 clearance, and this clearance rate was higher than that of WT M1 GAS strain SF370, probably due to excess amounts of FbaA compared to the physiological level (Fig. 5F). These results further illustrate that M1 GAS strain SF370-induced autophagy mainly depends on FbaA protein.

### The FbaA protein initiates autophagy upon binding of Fn to integrin α5β1 in epithelial cells.

**FIG 6:**
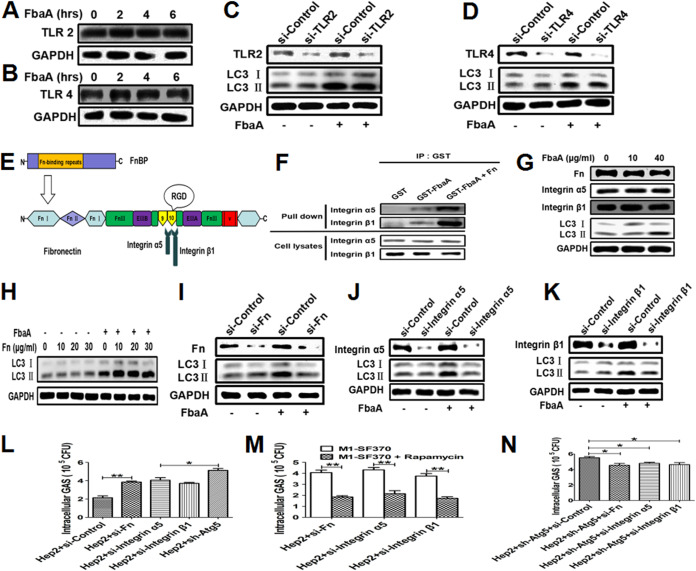
FbaA initiates autophagy upon Fn-mediated binding to integrin α5β1. (A and B) Western blot analysis of TLR2 (A) and TLR4 (B) expression in Hep2 cells stimulated with FbaA for the indicated times. (C and D) Expression of LC3II in Hep2 cells with and without TLR2- or TLR4-specific siRNA following FbaA stimulation. (E) Schematic representation of the Fn subunit. Fn contains domain types I, II, and III. FnI is the region critical for binding to FnBP; FnIII9 and FnIII10 contain motifs that interact with integrins. (F) Hep2 cells were transfected with GST-FbaA, GST-FbaA+Fn, or GST (as a control), and the integrin α5 or β1 chain was measured after being pulled down with anti-GST Ab. (G) Expression of Fn, integrin α5, integrin β1, and LC3II in Hep2 cells stimulated with FbaA at the indicated concentrations, as analyzed by Western blotting. (H) Western blot analysis of LC3II expression in Hep2 cells stimulated with different concentrations of Fn and mock-FbaA or FbaA. (I to K) Expression of LC3II in Hep2 cells with or without specific siRNA against Fn, integrin α5, or integrin β1 following FbaA stimulation, as analyzed by Western blotting. (L and M) Fn, integrin α5, and integrin β1 knockdown cells were pretreated without (L) or with (M) rapamycin for 0.5 h and then infected with M1 GAS strain SF370 for 6 h. Numbers of CFU of M1 GAS strain SF370 were counted in the cells. (N) M1 GAS strain SF370-infected sh-Atg5-Hep2 cells with specific siRNA against Fn, integrin α5, or integrin β1. Numbers of CFU of M1 GAS strain SF370 were counted in the cells. *, *P *< 0.05.

Toll-like receptors (TLRs) are major innate immunosensors of various pathogen-associated molecular patterns (PAMPs) (27). Among them, TLR2 and TLR4 play crucial roles in the activation of innate immune cells upon GAS infection. There is increasing evidence that TLRs are closely related to autophagy induction. To explore the roles of TLR2 and TLR4 in FbaA-induced autophagy, we first determined the expression levels of TLR2 and TLR4 in Hep2 cells stimulated with FbaA. The results showed negligible change in TLR2 or TLR4 expression in the cells with or without FbaA treatment (Fig. 6A and B). Furthermore, we knocked down TLR2 and TLR4 with short interfering RNA (siRNA) and then evaluated the effects on LC3II expression. The expression of FbaA-induced LC3II was not affected by TLR2 or TLR4 knockdown (Fig. 6C and D), indicating that neither TLR2 nor TLR4 is involved in FbaA-induced autophagy. It has been reported that GAS can invade host cells by binding to Fn. Fn is formed by the cross-linking of two subunits through a disulfide bond at the C termini. Each subunit of Fn has several structural domains, including three repetitive modules (module structures), 12 fibronectin type I repeat sites (FnI), 2 fibronectin type II repeat sites (FnII), 15 to 17 fibronectin type III repeat sites (FnIII), two alternative splicing sites (EIIIA and EIIIB), and one variable region (V) (28). Additionally, Fn is the major glycoprotein in the extracellular matrix and basement membrane, and it binds to a variety of pathogenic proteins (fibronectin binding protein, FnBP). FnBP interacts with the FnI module by binding the repeat domain near the N terminus, triggering a conformational change in Fn and exposing the concealed integrin binding site (Arg-Gly-Asp, RGD) in the 10th FnIII module (29). Integrin α5β1 is the major receptor that can be bound by Fn, and the FnBP-Fn complex binds to integrin α5β1 with a bond strength much greater than that of the classical Fn-integrin bond (Fig. 6E). Our results showed that, together, the FbaA protein and Fn bind more strongly to integrin α5β1 than does FbaA alone (Fig. 6F). Fn can be produced and secreted by epithelial cells, and integrin α5β1 is usually expressed on the surface of the epithelium and endothelial cells. To verify whether FbaA-induced autophagy affects the expression of Fn and integrin α5β1, Hep2 cells were stimulated with low (10 μg/ml) and high (40 μg/ml) doses of the FbaA protein, and then the levels of Fn and integrin α5β1 were measured. The expression of Fn and integrin α5β1 was not altered after FbaA treatment (Fig. 6G). Although the Fn protein had no effect on LC3 expression, when FbaA protein and Fn were added simultaneously, the expression of LC3II was increased significantly (Fig. 6H). Subsequently, we knocked down Fn or integrin α5β1 in Hep2 cells, stimulated the cells with FbaA protein, and then measured the LC3 expression. Knocking down Fn or integrin α5β1 did not alter LC3II expression in the cells without FbaA stimulation. However, the expression of LC3II was reduced in the Fn or the integrin α5β1 knockdown cells stimulated with FbaA protein compared to that with the siRNA-Control group (Fig. 6I to K), suggesting that the FbaA protein interacts with Fn and then binds to integrin α5β1 to initiate autophagy. To confirm their functional consequences, we used the WT M1 GAS strain SF370 to infect Hep2 cells, siRNA-Fn knockdown cells, siRNA-integrin α5 knockdown cells, and siRNA-integrin β1 knockdown cells and found that the number of viable bacteria was increased in the Fn, integrin α5, and integrin β1 knockdown cells compared to the number in control cells (Fig. 6L). However, M1 GAS strain SF370 survival was highest in the sh-Atg5 cells, suggesting that neither Fn nor integrin α5β1 was the only pathway to induce autophagy (Fig. 6L). Inducing autophagy with rapamycin also inhibited the growth of intracellular bacteria, indicating that autophagy was not effectively induced in the Fn or integrin α5β1 knockdown cells (Fig. 6M). Fn or integrin α5β1 was knocked down in the sh-Atg5 cells and then infected with WT M1 GAS strain SF370. The results showed that both Fn and integrin α5β1 knockdown cells had fewer viable bacteria than the control cells (Fig. 6N). The explanation for this outcome might be that Fn- and integrin α5β1-induced autophagy scavenging for GAS was lost in sh-Atg5 cells, but Fn- and integrin α5β1-mediated M1 GAS strain SF370 invasion was reduced. Taken together, these data demonstrate that FbaA induces autophagy dependent on its interactions with the Fn protein and membrane integrin α5β1.

### Induction of autophagy by integrin α5β1 and the FbaA-Fn complex is mediated through the mTOR-ULK1–Beclin-1 signaling pathway and Rab7 recruitment.

**FIG 7:**
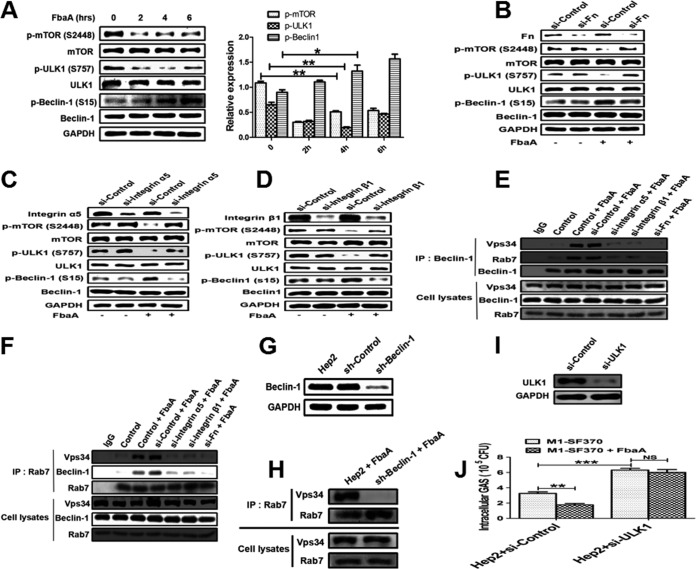
FbaA induces autophagy by activating mTOR-ULK1–Beclin-1 signaling and the formation of Beclin-1–Vps34-Rab7 complexes. (A) Western blot showing the expression of mTOR1, ULK1, Beclin-1, p-mTOR (S2448), p-ULK1 (S757), and p-Beclin-1 (S15) in Hep2 cells stimulated with FbaA for the indicated times. *, *P *< 0.05; **, *P *< 0.01. (B to D) The expression of mTOR1, ULK1, Beclin-1, p-mTOR (S2448), p-ULK1 (S757), and p-Beclin-1 (S15) in Hep2 cells treated with specific siRNAs targeting Fn, integrin α5, and integrin β1 after FbaA stimulation, as determined by Western blotting. (E and F) Vps34, Beclin-1, and Rab7 in Hep2 cells, Fn knockdown, and integrin α5β1 knockdown cells and the control cells in the presence or absence of FbaA, as determined by co-IP. Endogenous Vps34, Beclin-1, and Rab7 levels were also measured by Western blotting. (G and H) The Rab7 complexes immunoprecipitated from Hep2 cells and Beclin-1 knockdown cells were different after stimulation with FbaA (G), and endogenous Vps34 and Rab7 levels were measured by Western blotting. (I) Hep2 cells and ULK1 knockdown cells were pretreated with or without purified FbaA protein (30 μg/ml) for 0.5 h and then infected with M1 GAS strain SF370 for 6 h. (J) Numbers of CFU of intracellular M1 GAS strain SF370 in the cells were counted. *, *P *< 0.05; **, *P *< 0.01; ***, *P *< 0.001; NS, not significant.

It is well-known that ULK1 protein kinase and Vps34 lipid kinase are key checkpoints for the initiation and maturation of autophagy (30). ULK1 is regulated by the nutrient and energy receptors mTORC1 and AMPK. mTORC1 is the center of signaling transduction in host cells during bacterial infection and involved in downstream signal transduction of integrins. ULK1 could directly phosphorylate Beclin-1 (S15) (31). To gain insight into the mechanisms of FbaA-induced autophagy, we examined the phosphorylation status of mTOR1, ULK1, and Beclin-1 in cells stimulated with the FbaA protein. The results showed that the expression levels of p-mTOR (S2448) and p-ULK1 (S757) were decreased, while the expression of p-Beclin-1 (S15) increased in a time-dependent manner (Fig. 7A). To determine whether the changes in phosphorylation of these proteins depended on Fn or integrin α5β1, we knocked down Fn and integrin α5β1 and remeasured the phosphorylated protein levels. While the knockdown of Fn or integrin α5β1 had no effect on the levels of p-mTOR (S2448), p-ULK1 (S757), or p-Beclin-1 (S15) in the cells without FbaA, the expression of p-mTOR (S2448) and p-ULK1 (S757) was inhibited when FbaA was added (Fig. 7B to D). Phosphorylation of Beclin-1 can activate Vps34 and produce phosphatidylinositol 3-phosphate (PI3P), which recruits Rab7; the latter is a member of the GTPase family and is primarily involved in the transport of vesicles and the maturation of autophagosomes. Rab7 is a required component for GAS-induced autophagy in the formation of GcAVs (GAS-containing autophagosome-like vacuoles) (32). To determine whether Rab7 is involved in FbaA protein-induced autophagy, we first tested whether Rab7 and Vps34 interacted with each other. When cells were stimulated with FbaA protein, the anti-Beclin-1 antibody pulled down Vps34 and Rab7. These interactions disappeared when Fn or integrin α5β1 was knocked down in Hep2 cells, even after FbaA stimulation (Fig. 7E). In line with these data, the anti-Rab7 antibody pulled down Vps34 and Beclin-1 only in cells treated with FbaA but not in cells with integrin α5β1 or Fn being knocked down (Fig. 7F). In addition, knocking down Beclin-1 in Hep2 cells abolished the interaction between Rab7 and Vps34 (Fig. 7G and H). In ULK1 knockdown cells, the survival rate of M1 GAS strain SF370 was found to be higher, and the ability of FbaA to induce autophagy was also abrogated, indicating that M1 GAS strain SF370 was targeted for autophagy but not LC3-associated phagocytosis (Fig. 7I and J). Taken together, our data demonstrate that the interactions between FbaA, Fn, and integrin α5β1 protein activate the downstream signaling pathway mTOR-ULK1, which phosphorylates Beclin-1 and, thus, activates Vps34 and recruits Rab7 to promote the initiation of autophagy.

### Autophagy induced by FbaA and integrin α5β1 contributes to the clearance of invading M1 GAS strain SF370 *in vivo*.

**FIG 8:**
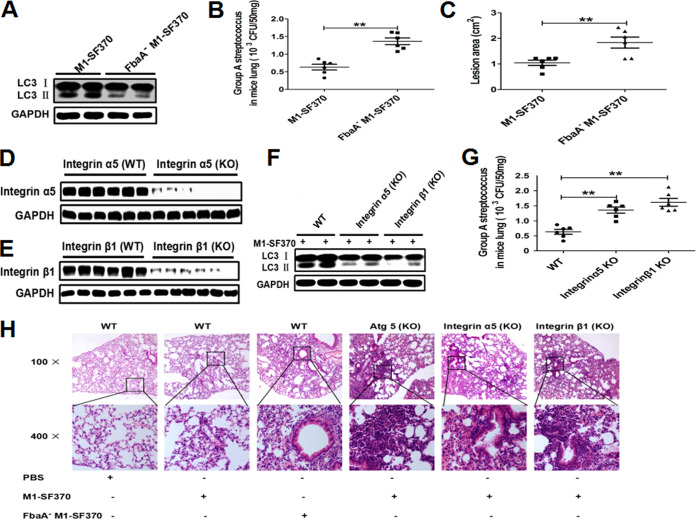
FbaA-initiated autophagy via integrin α5β1 enhances M1 GAS strain SF370 clearance in the lungs of mice. (A and B) Mice were infected with WT M1 GAS strain SF370 or FbaA^−^M1 GAS strain SF370 (3 × 10^8^ CFU) intranasally. Results of the analysis of LC3II expression by Western blotting and viable bacteria, in CFU, in the lungs of GAS-infected mice. (C) The mice were inoculated with WT M1 GAS strain SF370 and FbaA^−^M1 GAS strain SF370 in air pouches. Forty-eight hours after infection, the areas with skin lesions were measured by ImageJ software. **, *P *< 0.01. (D to H) WT, integrin α5-KO (D), and integrin β1-KO (E) mice were infected with WT M1 GAS strain SF370 (3 × 10^8^ CFU). The expression levels of integrin α5, integrin β1, and LC3II were analyzed with respective antibodies (F), and the CFU of M1 GAS strain SF370 in the lungs were counted (G). Lung pathology was determined based on hematoxylin-and-eosin-stained sections (H). Data are presented as the means ± SD from experiments with at least 6 mice per group. **, *P *< 0.01.

To verify whether FbaA mediates autophagy induction *in vivo* and determine its functional consequence on bacterial clearance, BALB/c mice were challenged with WT M1 GAS strain SF370 and FbaA^−^M1 GAS strain SF370 strains, and then the expression of LC3II and the survival of viable M1 GAS strain SF370 was evaluated. Twenty-four hours after infection, the expression of LC3II was reduced in lungs of the mice infected with FbaA^−^M1 GAS strain SF370 compared with that in mice infected with WT M1 GAS strain SF370 (Fig. 8A). The number of viable bacteria in the lungs of mice infected with FbaA^−^M1 GAS strain SF370 was significantly higher than it was in the lungs of the mice infected with WT M1 GAS strain SF370 (Fig. 8B). The lung pathology was also more severe in the mice with FbaA^−^M1 GAS strain SF370 infection than it was in those with WT M1 GAS strain SF370 infection (Fig. 8H). These data suggest that the reduced autophagy levels in the lungs of mice infected with FbaA^−^M1 GAS strain SF370 contribute to the increased bacterial loads and severe pathology. This finding also indicates that FbaA^−^M1 GAS strain SF370 induced a low level of autophagy, which led to low levels of FbaA^−^M1 GAS strain SF370 clearance. M1 GAS strain SF370 can infect not only lungs but also skin, causing skin damage. Analysis of skin lesions revealed a larger lesion area in the FbaA^−^M1 GAS strain SF370-infected mice than in the WT M1 GAS strain SF370 group at 48 h after challenge (Fig. 8C; see also Fig. S1 in the supplemental material). These results may be explained by the lack of FbaA and the inhibited autophagy, leading to a greater FbaA^−^M1 GAS strain SF370 load in skin epithelial cells than that of the WT M1 GAS strain SF370 group. Although FbaA^−^M1 GAS strain SF370 lacks the FbaA protein, the M protein is intact on the surface of FbaA^−^M1 GAS strain SF370 (the M protein, known as the main virulence factor of GAS). In addition, these results indicate that the FbaA protein is an important protein for M1 GAS strain SF370-induced autophagy *in vivo*.

10.1128/mBio.00771-20.1 FIG S1 Skin lesions of mice infected with WT M1 GAS strain SF370 or FbaA^−^M1 GAS strain SF370. The mice were injected subcutaneously with 1 ml of air to form an air pouch and then inoculated with 3 × 10^8^ CFUs of WT M1 GAS strain SF370, FbaA^−^M1 GAS strain SF370, or PBS as a control. After 48 h, the skin lesion areas were measured by ImageJ software. The arrowhead shows the location of the skin lesions. Download FIG S1, TIF file, 2.4 MB. Copyright © 2020 Wang et al. 2020 Wang et al. This content is distributed under the terms of the Creative Commons Attribution 4.0 International license.

To confirm the role of integrin α5β1 in inducing autophagy *in vivo*, we generated integrin α5 chain and β1 chain knockdown mice with CRISPR-Cas9 technology using adeno-associated viruses (Fig. 8D and E). WT M1 GAS strain SF370 was used to infect WT mice, integrin α5-KO mice, and integrin β1-KO mice. After 24 h, the expression of the LC3 protein and the number of viable bacteria in the lungs were measured. LC3II was nearly abolished in lungs of the integrin α5- and the β1-knockout mice (Fig. 8F), and the bacterial loads were increased (Fig. 8G). To define the role of autophagy in bacterial clearance, Atg5, integrin α5, and integrin β1-knockout mice were infected with WT M1 GAS strain SF370 or FbaA^−^M1 GAS strain SF370, and lung inflammation then was analyzed and compared. The WT mice infected with WT M1 GAS strain SF370 or FbaA^−^M1 GAS strain SF370 developed severe pulmonary inflammation characterized by lymphocytic infiltration, and FbaA^−^M1 GAS strain SF370 caused obvious thickening of the alveolar septum. Compared with WT mice infected with WT M1 GAS strain SF370, the Atg5-KO mice, integrin α5-KO mice, and integrin β1-KO mice showed increased infiltration of inflammatory cells, thickened alveolar septum, bleeding in the alveoli, and increased inflammatory lesions (Fig. 8H), indicating that M1 GAS strain SF370 induces autophagy through the interaction between FbaA protein and integrin α5β1 and subsequently facilitates the clearance of invaded M1 GAS strain SF370 from the host cells.

## DISCUSSION

Over the past few decades, the incidence of antibiotic-resistant bacteria has increased, which makes host-directed therapies (HDTs) increasingly important. Autophagy is an important innate defense mechanism and plays an essential role in identifying and degrading pathogens. Generally, most invasive bacteria (such as Salmonella enterica serovar Typhimurium, Mycobacterium tuberculosis, and Pseudomonas aeruginosa) (33, 34) can be targeted by autophagy for further clearance. Some bacteria can escape autophagy and survive, such as Shigella flexneri, Listeria monocytogenes (35), and Legionella pneumophila (36). Some bacteria can even use the nutrients produced by autophagy for self-proliferation, including Staphylococcus aureus (37), Coxiella burnetii, Helicobacter pylori (38), etc. However, the precise mechanism by which pathogens and hosts recognize each other and induce autophagy remains unknown.

Integrin is a transmembrane receptor that mediates connections between cells and the extracellular environment (e.g., extracellular matrix). Consisting of α and β subunits, integrin is expressed on the surface of almost all types of cells and plays a multifaceted role in cell fate, including platelet aggregation, angiogenesis, wound healing, and tumor cell migration (39, 40). Infection with pathogens often causes tissue damage and integrin receptor activation on the cell membrane, which transduces signals into the cells upon extracellular matrix components binding to integrin receptors. Fn is an important component of the extracellular matrix. Fn is a 230-kDa macromolecular glycoprotein widely distributed on the cell surface and in plasma (41) and consists of several domains that bind to collagen, fibrin, and cell membrane surface receptors. The most well-known binding domain is the Arg-Gly-Asp sequence (RGD) (42), which is the main site regulating cellular signaling after integrin receptor recognition. The surfaces of some pathogens also express Fn-binding protein (FnBP), which binds to Fn in the extracellular matrix, causing the Fn domain RGD to be exposed such that it can bind to integrin α5β1 on the cell surface (43). In this study, our data showed that after M1 GAS strain SF370 infects epithelial cells, the intracellular bacteria were cleared over time. However, this clearance did not occur in the Atg5^−/−^-Hep-2 cells. Similar clearance resistance was also shown in the Atg5-deficient mice. Furthermore, transmission electron microscopy (TEM) revealed a GAS-rich bilayer membrane structure formed in the cells and high levels of the LC3II protein, consistent with previous reports (44). These results suggest that M1 GAS strain SF370 induces autophagy in respiratory epithelial cells. Interestingly, heat-inactivated M1 GAS strain SF370 also induced autophagy, suggesting a role for M1 GAS strain SF370 structural proteins in autophagy induction. The FbaA and M proteins are known to bind to the cell surface receptor integrin α5β1 through Fn in the extracellular matrix. It has been reported that morphological transformation of the extracellular matrix can induce autophagy (45). However, our study demonstrates that Fn alone could not affect autophagy initiation. Both FbaA protein and M protein are Fn-binding proteins. Our data indicate that only FbaA protein is able to induce LC3II expression, decrease P62 expression, and aggregate GFP-LC3 spots in Hep2 cells either stimulated with purified proteins or infected with the M1 GAS strain SF370-deficient strain. In addition, pIB139-FbaA GAS, which was reintroduced with the FbaA gene, also could effectively induce autophagy. These results indicate that FbaA protein by itself can induce complete autophagic flux.

The reason that M protein could not induce autophagy may be due to differences in the Fn binding sites; the FbaA protein contains Fn-binding repeat regions that are absent from the M protein (25, 46). Thus, although both the FbaA and M proteins can bind to Fn, they probably initiate different signaling pathways (47, 48). In addition, the increased survival of the FbaA^−^M1 GAS strain SF370 in epithelial cells suggested that FbaA-induced autophagy contributes to M1 GAS strain SF370 elimination. To further explore the domain in FbaA protein responsible for autophagy induction, we constructed four functional peptides based on FbaA, and only the FbaA #68-161 peptide could induce autophagy. These results are consistent with our previous study showing that this region is the major domain for FbaA binding to Fn (extracellular matrix) or FH (serum proteins), respectively (25, 49).

Barnett et al. reported that the M1T1^5448^ clone of GAS degrades the autophagy adaptor proteins p62, NDP52, and NBR1 by secreting SpeB and replicates efficiently in Hep2 cells (10). The SpeB protease, an extracellular cysteine protease, is expressed in the late exponential to early stationary phases of growth. In our study, M1T1^2221^ GAS, but not M1 GAS strain SF370, can replicate efficiently in Hep2 cells, which is consistent with the results of Barnett et al. Whether the difference in survival between M1 GAS strain SF370 and M1T1^2221^ GAS in epithelial cells was related to SpeB expression level remains unknown (Fig. 1A). Our results showed that M1T1^2221^ GAS secreted high levels of SpeB, just like M1T1^5448^ GAS; however, M1 GAS strain SF370 hardly secreted it (see Fig. S2 in the supplemental material). Therefore, the difference between the two may be due to the high level of SpeB secreted by M1T1^2221^ GAS, which clears autophagy components from the surface of the M1T1 bacterium, avoids ubiquitylation and recognition by the host autophagy marker LC3 and ubiquitin-LC3 adaptor proteins NDP52, p62, and NBR1, and eventually leads to M1T1^2221^ GAS, but not M1 GAS strain SF370, not being killed by autophagy.

10.1128/mBio.00771-20.2 FIG S2 Western blot analysis of the SpeB expression by M1T1^2221^ GAS and M1 GAS strain SF370. The expression level of SpeB in supernatants of M1T1^2221^ GAS and M1 GAS strain SF370 were detected by Western blotting after bacteria were incubated for 6 h in THY medium. Download FIG S2, TIF file, 1.4 MB. Copyright © 2020 Wang et al. 2020 Wang et al. This content is distributed under the terms of the Creative Commons Attribution 4.0 International license.

Davies et al. screened a collection of 2,083 global GAS strains and found that the FbaA gene is only present in <10% of GAS strains (50), which was detected in strains M1, M2, M4, M9, M13, M22, M28, M44, M49, M60, M67, M75, M77, M79, M80, M82, M87, and M89. Our previous studies also showed that among different strains of the same serotype, the expression level of FbaA on the surface of the bacteria varies greatly, indicating the diversity of GAS strains and the differing virulence strategies present in the GAS strains. Therefore, it will be important to find different interactions between GAS strains and host cells. TLRs are important pathogen recognition receptors in mammalian cells. Whether TLRs mediate autophagy is debatable. Some reports show that TLRs control autophagy signaling. Sanjuan et al. observed that GFP-LC3 was recruited to phagosomes of the latex beads associated with various TLR ligands, but no typical dual membrane structure was observed (51). TLR2 and TLR4 are essential pattern receptors for the recognition of GAS. However, we did not observe any changes in TLR2 or TLR4 expression in the Hep2 cells stimulated with FbaA protein or in LC3II expression in TLR2 or TLR4 knockdown cells. Our data indicate that TLR2 and TLR4 do not participate in FbaA-induced autophagy. Surprisingly, we found that the levels of LC3II were greatly reduced in integrin α5β1 knockout cells, while the survival rate of intracellular bacteria was greatly increased, and these changes were reversed after the addition of the autophagy inducer rapamycin. Data from integrin α5β1 knockout mice also confirmed the *in vitro* findings. Thus, our data demonstrate that M1 GAS strain SF370-induced autophagy depends on the surface protein FbaA-Fn-integrin α5β1 both *in vitro* and *in vivo*.

Despite the central role of autophagy in the innate immune control of invading microorganisms, the characteristics and activation mechanisms of the interaction between integrin and autophagy remain unclear. Deuretzbacher et al. (52) and Gluschko et al. (53) reported that integrin β1 or αMβ2 mediates the phagocytosis of Yersinia enterocolitica or Listeria monocytogenes in macrophages, and then LC3 accumulates around bacterium-containing vacuoles or induces a noncanonical form of autophagy (LC3-associated phagocytosis [LAP]). Interestingly, Portillo et al. showed that Toxoplasma gondii infected host cells through integrin β1 and β3, thereby inducing FAK-Src-STAT3 signal transduction to prevent autophagy (54). It is unclear whether integrin can directly initiate the canonical autophagy of epithelial cells and, thus, not act as a phagocytic receptor of phagocytes that mainly mediates the phagocytosis of pathogens to induce noncanonical autophagy or suppress autophagy in cells. Our study demonstrated that pathogens preferentially initiate autophagy by utilizing FnBP to form complexes with Fn and integrin α5β1 in epithelial cells.

**FIG 9:**
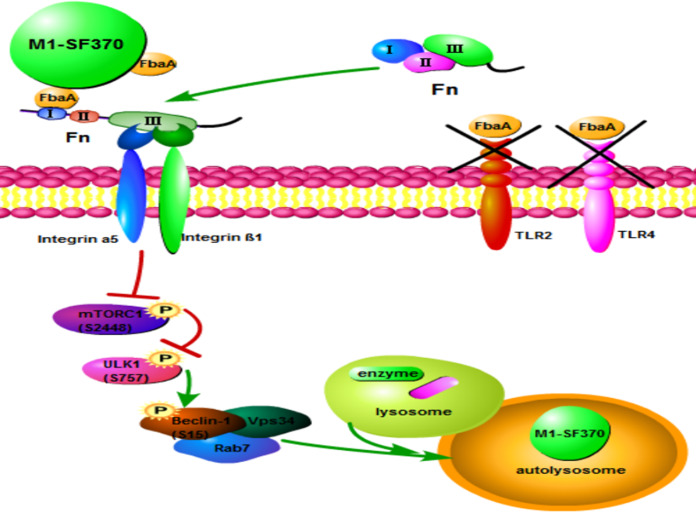
Schematic of autophagy induction by M1 GAS strain SF370 through the interaction with FbaA and the Fn-integrin α5β1 complex via the mTOR-ULK1–Beclin-1 pathway. The binding of FbaA, Fn, and integrin α5β1 leads to mTOR inactivation (S2448) and ULK1 dephosphorylation (S757) and then to phosphorylated Beclin-1 (S15), which activates VPS34 kinase to recruit Rab7, promoting autophagosome maturation.

The expression pattern of cell surface integrins determines the binding capability of integrin to components in the ECM and triggers different downstream signal events, which ultimately affect cellular immune responses. After integrin molecules bind to ECM components, the integrin aggregates and initiates specific intracellular signals through its cytoplasmic domain. For example, the FAK-Ras-mitogen-activated protein kinase signaling pathway can mediate cell adhesion and migration (55), and paxillin and talin activation mediates cytoskeletal rearrangement (56) and even regulate EGFR activity and alter the downstream enzyme activity, such as that of ERK, AKT, JNK, and Rho GTP (57, 58). Here, we report that the formation of the FbaA-Fn complex activates integrin α5β1, which deactivates mTOR and subsequently attenuates the inhibition of ULK1 activity through the dephosphorylation of Ser757. Our data demonstrate that FbaA-dependent integrin α5β1 induces autophagy through the classical mTOR-ULK1 autophagy signaling pathway. We found that FbaA-induced autophagy can be completely inhibited by 3-MA (a potent inhibitor of class III phosphatidylinositol 3-kinase [PI3K]). Therefore, we speculate that class III PI3K is involved in the process of autophagy induction. The production of phosphatidylinositol 3-phosphate (PI3P) by activated Vps34 (the only class III PI3K in mammals) is an early key event in the formation of autophagosomes (59, 60), and the primary role of PI3P is to recruit proteins that form the autophagosome membranes (61). Rab family proteins are important regulatory proteins in the formation of autophagosome membranes (62, 63). Among them, Rab7 can directly interact with Vps34 and plays a critical role in the formation of autophagosomes and autolysosomes (64, 65). As shown in Fig. 7E and F, Beclin-1, Vps34, and Rab7 interacted with each other after cells were stimulated with FbaA. Our study further showed that Rab7 did not interact with Vps34 in Beclin-1^−/−^ cells, suggesting that FbaA-induced autophagy depends on the Beclin-1 protein to act as a bridge between Rab7 and Vps34. Activated ULK1 directly phosphorylates Beclin-1 (S15), a finding consistent with the reports of Russell et al. (31). Furthermore, we found that when Fn, the integrin α5 chain, or the integrin β1 chain activity was knocked down with siRNA, the activity of mTOR was increased, the ULK1 phosphorylation at Ser757 was increased, the level of phosphorylated Beclin-1 (S15) was reduced, and, thus, Beclin-1, Vps34, and Rab7 could not interact with each other, eventually leading to the failure of FbaA-induced autophagy. This finding suggests that FbaA needs to bind to Fn to activate its receptor, integrin α5β1, which promotes the formation of the Beclin-1–Vps34-Rab7 complex and, thus, initiates autophagy (Fig. 9).

Cheng et al. found that M49 GAS induced LAP in endothelial cells through the activation of integrin β1 by secreting virulence factor SLO, which induces NOX2 recruitment and ROS production, thereby enhancing M49 GAS proliferation (66). Here, integrin β1 acts as the receptor for LAP induction. In addition to LAP, the researchers observed that M49 GAS infection also induced canonical autophagy in endothelial cells; however, it remains unclear which M49 GAS components play a role in autophagy induction. In this study, the formation of bilayer membrane vesicles (indicated by arrows in Fig. 1F and 7J) suggested that the process depends on ULK1 through canonical autophagy but not LAP. The initiation of autophagy was caused by the structural protein FbaA of M1 GAS strain SF370 rather than the secretory protein, forming a complex with Fn and integrin α5β1 to promote the formation of the Beclin-1–Vps34-Rab7 complex upon activation of the mTOR-ULK1 signaling pathway and, finally, leading to the elimination of M1 GAS strain SF370 in epithelial cells. Additionally, whether the activation of integrin α5β1 was spatially and directly coupled with the formation of autophagosomes needs further investigation. Although FbaA can induce high levels of autophagy in Hep2 cells, the data shown in Fig. 2C and D and 6L suggest that FbaA is not the only component influencing M1 GAS strain SF370-induced autophagy.

In summary, this study reveals a novel model for autophagy induction initiated in host cells after bacterial infection. Our results indicate that cell surface integrin α5β1 plays a decisive role in inducing autophagy by bacterial protein FbaA. As many pathogens can interact with ECM-related proteins to activate integrins, we speculate that integrin α5β1-initiated autophagy is a common event in epithelial cells for host defense against pathogen infection. Currently, integrin-related drugs have been successfully used in the treatment of tumors (67). This study provides new insights into the interaction pattern between pathogens and hosts and may provide a new strategy for developing host-directed autophagy to treat drug-resistant bacteria.

## MATERIALS AND METHODS

### Bacterial culture.

M1 GAS strain SF370 and other mutant GAS strains (FbaA^−^M1 GAS strain SF370 and M^−^M1 GAS strain SF370) were stored at –80°C in our laboratory. M1^2221^ GAS was provided by Jinquan Li. Cryopreserved GAS was inoculated onto blood agar plates at 37°C and incubated for 24 h. A single colony was transferred to 3 ml of Todd-Hewitt broth supplemented with 0.5% yeast extract (THY) at 37°C and shaken at 220 rpm overnight. Nonviable M1 GAS strain SF370 was prepared by heating bacteria to 70°C for 60 min.

### Cells.

The human laryngeal epithelial cell line Hep2 (obtained from The Institute of Basic Medical Sciences of the Chinese Academy of Medical Sciences, China) was cultured in RPMI 1640 medium (Gibco) supplemented with 100 U/ml penicillin and 100 U/ml streptomycin (Solarbio), 10 mM HEPES (AMRESCO 0511; Biosharp), and 10% fetal bovine serum (Biological Industries) at 37°C with 5% CO_2_ in an incubator.

### GAS infection.

Hep2 cells were seeded at 3 × 10^5^/well in 6-well plates containing RPMI 1640 medium without antibiotics. Hep2 cells were cocultured overnight with bacteria inoculated at a multiplicity of infection (MOI) of 100:1 for 2 h at 37°C with 5% CO_2_ in an incubator. To kill adherent extracellular bacteria, the cells were washed three times with PBS and then incubated with fresh RPMI 1640 containing 100 μg/ml gentamicin at 37°C for 2 h. Finally, the Hep2 cells were collected at specific time points for Western blot analysis.

### shRNA knockdown.

As previously described (68), briefly, Hep2 cells stably expressing specific short hairpin RNAs (shRNAs) against Atg5, Beclin-1, or luciferase were established by transduction with lentivirus. Lentiviral particles were packaged by transfecting 293T cells with pSIF-H1-copGFP shRNA expression lentivectors (System Biosciences) and packaging vectors using Lipofectamine 2000 according to the manufacturer’s instructions.

### siRNA.

Before transfection, Hep2 cells were seeded into 6-well plates. When the cells grew to 60% confluence, the Hep2 cells were transiently transfected with the corresponding siRNA by HiPerFect transfection reagent (30 nM). After 48 h, the silencing efficiency was determined with Western blotting. The following siRNAs were used: Beclin1 siRNA, sense, 5′GAUGAUGAGCUGAAGAGUGUUGAA3′; antisense, 5′UUCAACACUCUUCAGCUCAUCAUCC3′; TLR2 siRNA, sense, 5′AAAUCUGAGAGCUGCGAUATT3′; antisense, 5′UAUCGCAGCUCUCAGAUUUTT3′; TLR4 siRNA, sense, 5′CGAUGAUAUUAUUGACUUAT3′; antisense, 5′UAAGUCAAUAAUAUCAUCGTT3′; Fn siRNA, sense, 5′GUCCUGUCGAAGUAUUUAUTT3′; antisense, 5′AUAAAUACUUCGACAGGACTT3′; integrin α5 siRNA, sense, 5′CACCCGAAUUCUGGAGUAUTT3′; antisense, 5′AUACUCCAGAAUUCGGGUGTT3′; integrin β1 siRNA, sense, 5′GCACCAGCCCAUUUAGCUATT3′; antisense, 5′UAGCUAAAUGGGCUGGUGCTT3′; ULK1 siRNA, sense, 5′CGCGGUACCUCCAGAGCAATT3′; antisense, 5′UUGCUCUGGAGGUACCGCGCG3′.

### Expression and purification of the M protein, FbaA protein, and GST-FbaA protein.

Purified M protein, FbaA protein, and glutathione *S*-transferase (GST)-FbaA protein were conserved in our laboratory. The purification steps of these proteins were previously described (23, 25); briefly, expression of the GST-M and GST-FbaA proteins was performed in GEX2T/fbaA- or GEX2T/M-transformed E. coli BL21 following induction with isopropyl-β-d-thiogalactopyranoside (IPTG) (0.1 mM; Merck). The GST-FbaA and GST-M proteins were purified by glutathione Sepharose 4B (GE Healthcare). To rule out the possibility of contamination in the purified protein preparations, polymyxin B columns were used (Thermo Scientific). For the removal of the GST tag, thrombin was used, as needed, to cleave GST from the protein according to the manufacturer’s instructions (GE Healthcare).

### Electroporation.

To prepare FbaA^−^M1 GAS strain SF370 competent cells, the bacteria were harvested in the early-log to mid-log phase (optical density at 600 nm of 0.3 to 0.4) and washed twice with precooled deionized water at 4°C and 3,500 rpm for 7 min. They then were washed twice with deionized aqueous solution containing 10% glycerol at 4°C and 3,500 rpm for 7 min. Using Gene Pulser Xcell (Bio-Rad), the pIB139-FbaA plasmid with the apramycin resistance gene was transferred into FbaA^−^M1 GAS strain SF370 by electroporation at 1.8 kV/mm, 25 μF, and 400 Ω. After incubation at 37°C for 2 h, the competent cells were spread on THY agar plates containing apramycin and cultured for 24 to 48 h until single colonies appeared.

### Western blotting.

Cells were collected and lysed with radioimmunoprecipitation assay lysis buffer (P0013; Beyotime) containing phenylmethylsulfonyl fluoride (BL507A; Biosharp) and phosphatase inhibitors (P1260; Solarbio) on ice for 30 min. The components in the supernatant (denatured at 100°C in sample buffer) were separated using SDS-PAGE and transferred to 0.45-μm or 0.22-μm polyvinyl difluoride (PVDF) membranes (IPVH00010; Millipore). The PVDF membranes then were blocked with 5% nonfat milk for 1 h and incubated overnight with primary antibodies at 4°C. Subsequently, the PVDF membranes were washed with Tris-buffered saline-Tween 20 and incubated with the corresponding secondary antibody for 1 h. Finally, the proteins were detected with Western Lightning plus-ECL reagent (NEL104001EA; PerkinElmer) and detected with a Synoptics Syngene bioimaging instrument (R114075; Synoptics).

### Antibodies and reagents.

For Western blot analysis, the following antibodies were used: anti-LC3B (2775; Cell Signaling Technology), anti-SQSTM1/P62 (5114; Cell Signaling Technology), anti-ATG7 (DF6130; Affinity Biosciences), anti-ATG5 (CY5766; Abways), anti-fibronectin (ab32419; Abcam), anti-integrin α5 (CY5979; Abways), anti-integrin β1 (CY5469; Abways), anti-phospho-Beclin-1 (Ser15) (84966; Cell Signaling Technology), anti-BECN1 (ab207612; Abcam), anti-TLR2 (AF7002; Affinity Biosciences), anti-TLR4 (AF7017; Affinity Biosciences), anti-CST (AB0055; Abways), anti-Rab7 (9367; Cell Signaling Technology), anti-phospho-MTOR (Ser2448) (AF3308; Affinity Biosciences), anti-MTOR (AF6308; Affinity Biosciences), anti-phospho-ULK1 (Ser757) (6888; Cell Signaling Technology), anti-ULK1 (8054; Cell Signaling Technology), and anti-glyceraldehyde-3-phosphate dehydrogenase (GAPDH) (5174; Cell Signaling Technology). Horseradish peroxidase-labeled goat anti-rabbit (ASS1009; Abgent) secondary antibodies were used. The transfection reagents were Lipofectamine 2000 (11688-019; Invitrogen) and HiPerFect (Y5-301705; Qiagen). The immunoprecipitation (IP) reagent was included in the Pierce classic magnetic IP/co-IP kit (88804; Thermo Scientific).

### Immunofluorescence.

Hep2 cells were seeded in 12-well plates and transfected with pBABEpuro-EGFP-LC3 plasmids using Lipofectamine 2000 for 24 h. The cells then were stimulated by GAS or FbaA protein for 6 h. After washing with PBS, the cells were fixed in 4% paraformaldehyde for 20 min and blocked in 1% bovine serum albumin for 1 h. The cells then were incubated with the corresponding primary antibody overnight at 4°C. After four rinses, the secondary antibody was used at a suitable concentration for 1 h. 4′,6-Diamidino-2-phenylindole (DAPI) was used to stain the cell nuclei. Finally, the cells were visualized by an Olympus confocal fluorescence microscope.

### IP.

Immunoprecipitation assays were performed according to the instructions of the Pierce classic magnetic IP/co-IP kit. Briefly, the cells were lysed in specific buffer on ice for 30 min. The supernatant protein then was incubated overnight with the corresponding antibody on a rotator at 4°C. The next day, Pierce protein A/G beads were washed with specific buffer three times. The supernatant-antibody mixture and the beads then were coincubated on a rotator at room temperature for 2 h, washed with lysis buffer and PBS, and boiled for 10 min. The samples were subjected to SDS-PAGE and Western blot analysis, and the target proteins were detected using the corresponding antibodies.

### Transmission electron microscopy.

Autophagosomes induced by M1 GAS strain SF370 in the Hep2 cells were analyzed under a Hitachi 7500 transmission electron microscope, as previously described, with a small modification (68). Briefly, Hep2 cells were seeded in 6-well plates and infected with M1 GAS strain SF370 at an MOI of 100 for 2 h. After washing with PBS, the cells were incubated for an additional 6 h. The cells then were trypsinized and collected by centrifuging at 1,000 rpm for 5 min and fixed overnight with 2.5% glutaraldehyde in 0.1 M sodium cacodylate buffer at 4°C. Subsequently, the fixed cells were postfixed with 1% osmic acid, dehydrated stepwise with ethanol, and embedded in epoxy resin. Ultrathin sections were cut using a Leica ultramicrotome and stained with uranyl acetate and lead citrate. The cells were imaged using a Hitachi 7500 transmission electron microscope at an 80-kV acceleration voltage. The procedure described above was performed by the Electron Microscope Center of Hebei Medical College.

### H&E staining.

The histology of the GAS-infected mouse lung tissue was analyzed using hematoxylin-eosin staining (H&E), as described previously (69). In brief, the mice were anesthetized using isoflurane in a biosafety cabinet. M1 GAS strain SF370 was delivered intranasally (i.n.) (3 × 10^8^ CFU in 50 μl of PBS). After 24 h, the mouse lungs were fixed with 4% polyformaldehyde, embedded in paraffin, cut into 5-μm-thick sections, and stained with H&E. The slides were examined by light microscopy.

### Cell and lung CFU determination.

Hep2 cells were infected with M1 GAS strain SF370 (MOI, 100) for 6 h at 37°C in 5% CO_2_–95% air. After washing with PBS three times, the infected monolayers were dispersed by the addition of 0.25% trypsin and then lysed by dilution with sterile water. The number of bacterial CFU released from the lysed cells was determined by plating lysates on blood agar at 37°C for 24 h.

As shown previously, the mice were infected with M1 GAS strain SF370, and then the lungs were aseptically isolated and weighed. Subsequently, the lungs were homogenized in RPMI 1640 (containing antibiotics) and lysed as described above with sterile water. The number of bacterial CFU released from the lysed lung cells was determined by plating the lysates on blood agar at 37°C for 24 h.

### Animal experiments.

BALB/c mice (8-week-old females) were purchased from the Experimental Animal Center of Hebei Medical University. The number of the Laboratory Animal Quality Certificate is 1811002. All experimental procedures were performed in compliance with institutional animal welfare guidelines and were carried out according to the criteria outlined in the *Guide for the Care and Use of Laboratory Animals* (70) and with approval of the Animal Care and Use Committee of Hebei Medical University. The mice were maintained in an animal facility (temperature-controlled individual ventilated cages) under a 12-h light/dark cycle and were fed standard chow and sterile tap water.

**(i) Air pouch model of infection.** After being anesthetized with pelltobarbitalum natricum, the mice were injected subcutaneously with 1 ml of air to form an air pouch and then inoculated with 0.5 ml of PBS (containing 3 × 10^8^ CFU of GAS). After 48 h, the skin lesion areas were measured by ImageJ software.

**(ii) AAV6 transmission into the lungs of mice.** The vectors (containing CRISPR-scrambled single guide RNA all-in-one adeno-associated virus 6 [AAV6] with saCas9, integrin β1 CRISPR all-in-one AAV6 with saCas9, and integrin α5 CRISPR all-in-one AAV6 with saCas9) were constructed by Applied Biological Materials, Inc. The mice were anesthetized using isoflurane in a biosafety cabinet. The vectors were delivered intranasally (1 × 10^11^ viral genome copies/mouse). After 3 weeks, the mouse lungs were isolated for Western blot verification.

**(iii) Atg5-KO mice.** Atg5^flox/flox^ mice were provided by the RIKEN BRC through the National Bio-Resource Project of the MEXT, Japan, and kindly given by Quan Chen, Institute of Zoology, Chinese Academy of Sciences (71). Specific Atg5^flox/cre^ mice were generated by mating Atg5^flox/flox^ mice with Sftpc-cre mice (purchased from ViewSolid Biotech, Beijing, China). Intraperitoneal injection of tamoxifen (Sigma‐Aldrich) was administered to inducible cre-driver lines. The injection dose was determined by weight using approximately 75 mg/kg of body weight tamoxifen. The tamoxifen dose was administered via intraperitoneal injection once every 24 h for a total of 5 consecutive days. For Cre characterization work, there is a 7-day waiting period between the final injection and necropsy/histological analysis.

### Statistical analysis.

SPSS statistical software (version 16.0) was used for statistical analysis.

The data are expressed as the means ± standard deviations (SD). The significance between two groups was determined using a *t* test. A *P* value of <0.05 (*) was considered significant. All experiments were performed in triplicate or more replicates.

## Note Added after Publication

The corresponding author information was changed after the initial publication (9 June 2020). The article was updated online on 16 July 2020.
