# Erratum for Wang et al., “Integrin α5β1, as a Receptor of Fibronectin, Binds the FbaA Protein of Group A *Streptococcus* To Initiate Autophagy during Infection”

**DOI:** 10.1128/mBio.01729-20

**Published:** 2020-07-21

**Authors:** Jiachao Wang, Meiqi Meng, Miao Li, Xiaofei Guan, Jianguo Liu, Xue Gao, Qingqing Sun, Jinquan Li, Cuiqing Ma, Lin Wei

**Affiliations:** a Department of Immunology, Key Laboratory of Immune Mechanism and Intervention on Serious Disease in Hebei Province, Hebei Medical University, Shijiazhuang, China; b Departments of Internal Medicine & Molecular Microbiology and Immunology, Saint Louis University School of Medicine, St. Louis, Missouri, USA; c State Key Laboratory of Agricultural Microbiology, College of Food Science and Technology, Huazhong Agricultural University, Wuhan, Hubei, China

## ERRATUM

Volume 11, no. 3, e00771-20, 2020, https://doi.org/10.1128/mBio.00771-20. The corresponding author statement should read as shown below. The article has been updated online:

“Address correspondence to Lin Wei, weilin@hebmu.edu.cn, or Cuiqing Ma, macuiqing@hebmu.edu.cn.”

