# Supplementary figures and images for: Integrin α5β1, as a Receptor of Fibronectin, Binds the FbaA Protein of Group A Streptococcus To Initiate Autophagy during Infection
Source: mBio. 2020 Jul 16;11(3):e00771-20. doi: 10.1128/mBio.00771-20 (PMC7371361; doi:10.1128/mBio.00771-20)

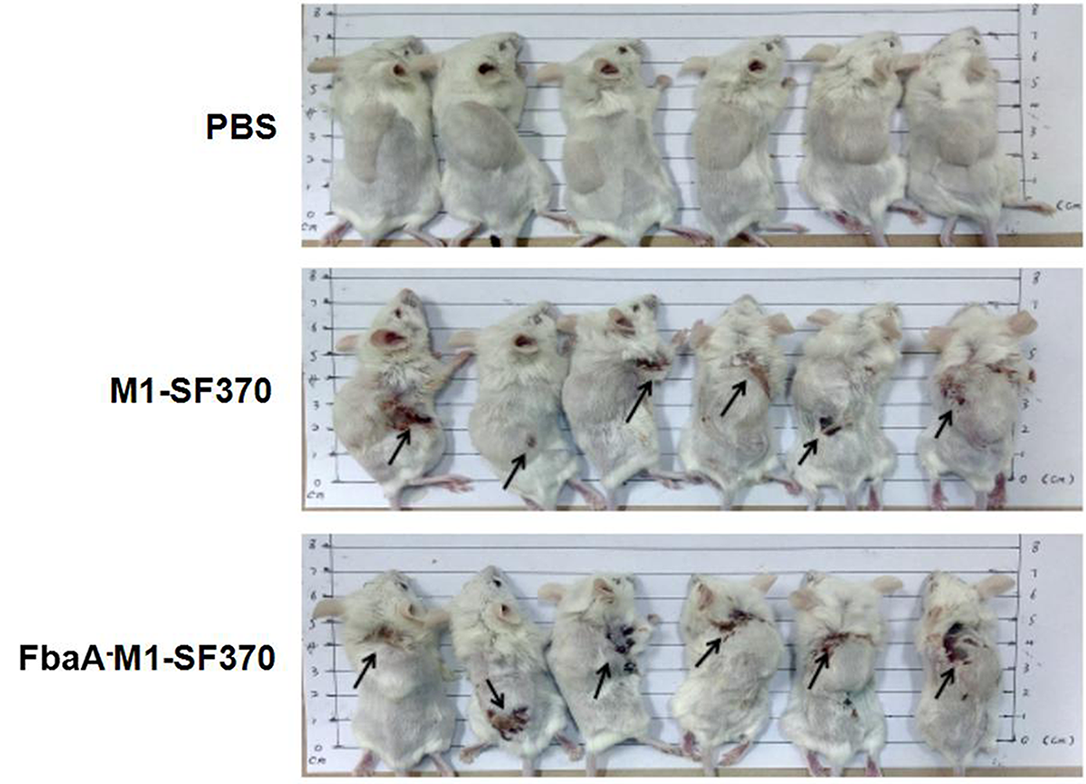

Supplement: FIG S1 [file mBio.00771-20-sf001.tif]

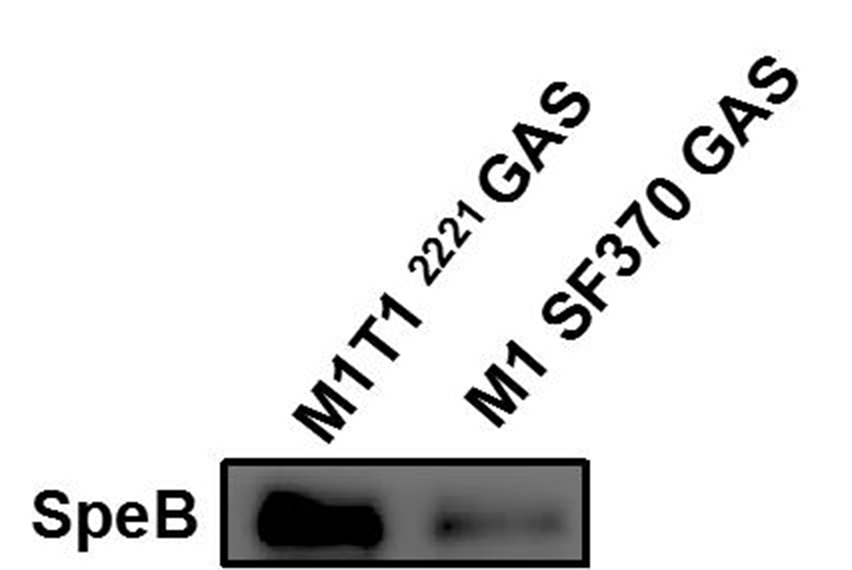

Supplement: FIG S2 [file mBio.00771-20-sf002.tif]
